# Effects of cassava polysaccharides on gut microbiome, intestinal barrier and macrophage activation

**DOI:** 10.3389/fimmu.2026.1874777

**Published:** 2026-07-02

**Authors:** Houmei Yu, Qingqun Yao, Liming Lin, Chunping Jian, Peixu Du, Jinquan Zhang, Yusi Fang, Mengying Liu, Qinfei Wang, Zhenwen Zhang

**Affiliations:** 1 Tropical Crops Genetic Resources Institute, Chinese Academy of Tropical Agricultural Sciences/National R&D Centre for Potato Processing, Haikou, Hainan, China; 2 Key Laboratory of Ministry of Agriculture for Germplasm Resources Conservation and Utilization of Cassava, Haikou, Hainan, China; 3 Key Laboratory of Tropical Crops Germplasm Resources Genetic Improvement and Innovation of Hainan Province, Haikou, Hainan, China; 4 Hainan State Farms Academy of Sciences Group Co., Ltd., Sanya, Hainan, China

**Keywords:** cassava polysaccharides, crude polysaccharide of cassava root, intestinal barrier, RAW264.7 macrophages, short-chain fatty acids

## Abstract

CPs possess considerable bioactive potential, yet their underlying immunomodulatory mechanisms remain incompletely elucidated. In the present work, CPCR were extracted from fresh cassava tubers and further separated into five purified polysaccharide fractions (CP1–CP5) with distinct monosaccharide profiles and molecular weights. Systematically investigated the immunomodulatory capacities of CPCR and its purified fractions via *in vivo* assays using Cy-induced immunosuppressed mice and *in vitro* tests on RAW264.7 murine macrophages. Multiple readouts were quantified, including gut microbial community structure, fecal SCFAs concentrations, intestinal tight junction protein expression, serum anti-inflammatory cytokine levels, as well as macrophage proliferation, phagocytic activity and inflammatory mediator release. *In vivo* data demonstrated that CPCR reshaped gut microbiota homeostasis by selectively enriching beneficial commensal genera and families linked to intestinal health, namely *Muribaculaceae, Bacteroides, Alloprevotella*, and *Prevotellaceae*. Enrichment of these probiotic taxa boosted intestinal SCFAs production; notably, fecal acetic acid concentration reached 141.0 mg/g following CPCR intervention, significantly exceeding levels measured in both normal control and Cy-induced immunosuppressed groups. Moreover, CPCR robustly upregulated the expression of intestinal barrier proteins ZO-1, occludin and Claudin-1, facilitating the repair and preservation of intestinal epithelial integrity. Serum cytokine profiling revealed prominent elevations in the anti-inflammatory mediators IL-2, IL-4 and IL-10 upon CPCR administration. Structural characterization of isolated subfractions revealed stark compositional disparities: CP1 predominantly consisted of 97% glucose with a molecular weight of 3 kDa, while CP2 contained 31.1% glucose, 20% galactose and 15.2% arabinose with a molecular weight of 62.4 kDa, this represents a preliminary structural characterization of the polysaccharide fractions. The results demonstrated that all CPs fractions could enhance immune cell activity, including phagocytic capacity and anti-inflammatory cytokine secretion. In summary, this study demonstrates that CPs exert immunostimulatory effects through dual pathways: direct activation of macrophage immune function and indirect regulation of gut microbiota-intestinal barrier homeostasis. Our results support the translational potential of CPs as bioactive functional food ingredients for immune regulation.

## 1 Introduction

Natural polysaccharides have demonstrated broad application prospects in immunomodulation, antioxidant activity, and antitumor effects due to their unique bioactivities (1–3). Among these bioactivities, their immunomodulatory properties are of particular clinical significance, as a robust and well-regulated immune system serves as a defense mechanism against various pathological conditions (4, 5). Immunodeficiency states have been closely associated with increased susceptibility to bacterial, viral, and fungal infections, as well as elevated risks of malignancies (6). Unlike synthetic immunomodulators, plant-derived polysaccharides offer the advantage of regulating immune function through multiple pathways and multi-level mechanisms with minimal adverse effects (7).

The immunomodulatory roles of polysaccharides are multifaceted, involving both direct cellular activation and indirect regulatory pathways. These compounds can directly activate various immune cell populations, including T cells, B lymphocytes, macrophages, and natural killer cells, while simultaneously stimulating the complement system and promoting cytokine secretion (7, 8). Key cytokines such as interleukin-2 (IL-2), IL-6, and tumor necrosis factor-α (TNF-α) play crucial roles in modulating the immune microenvironment and maintaining immunological homeostasis (9–11). Recent evidence has also highlighted the importance of the gut-immune axis, where polysaccharides influence systemic immunity through modulation of intestinal microbiota composition and metabolite production (12).

In immunological research, macrophages serve as key targets for immunomodulation studies (13), with the RAW264.7 murine macrophage cell line representing a well-established *in vitro* model for evaluating immunomodulatory compounds (14). For *in vivo* studies, cyclophosphamide (Cy) is a widely used chemotherapeutic and immunosuppressive agent (15). It suppresses both cellular and humoral immune responses and leads to gut microbiota dysbiosis and increased intestinal permeability (16). Consequently, Cy has been commonly employed in preclinical research to induce experimental immunosuppression, thereby providing a relevant model for evaluating immunoenhancing interventions (17).

Cassava (*Manihot esculenta* Crantz) is a pivotal agricultural commodity in tropical regions, extensively cultivated across Africa, Southeast Asia, and Latin America. It serves as a staple food crop rich in both starch and non-starch polysaccharides (NSPs). Among these components, NSPs exhibit remarkable biological activities attributable to their diverse monosaccharide compositions (18, 19). Previous studies have identified various bioactive polysaccharides in cassava roots, demonstrating significant potential in enhancing exercise endurance, as well as exerting antioxidant and hepatoprotective effects in animal models (20–22). Notably, while these established bioactivities imply that cassava polysaccharides (CPs) may interact with immune-related pathways, their specific immunomodulatory mechanisms remain largely uncharacterized.

The combination of high safety profile, abundant availability, and low production costs makes CPs particularly attractive for developing immunomodulatory functional materials. Although CPs exhibit therapeutic potential in anti-fatigue, anti-oxidation, and anti-tumor effects (21–23), systematic studies investigating their immunomodulatory mechanisms remain limited. Specifically, the dual role of CPs in direct immune cell activation and indirect regulation through gut microbiota modulation has not been comprehensively characterized.

In this work, we first extracted Crude polysaccharide from cassava roots (CPCR) using ultrasonic-assisted enzymatic hydrolysis and evaluated its *in vivo* immunomodulatory effects in Cy-induced immunosuppressed mice by detecting serum indices, intestinal barrier function, gut microbiota, and short-chain fatty acids (SCFAs). Subsequently, we purified CPCR into five fractions and characterized their structural features. Finally, we investigated the *in vitro* immunomodulatory mechanisms of all CPs fractions using RAW264.7 macrophages. This study aims to provide new ideas for the utilization of CPs as immunomodulatory agents.

## 2 Materials and methods

### 2.1 Materials, reagents and experimental animals

The roots of ‘South China 9′ (SC9) cassava were provided by the National Cassava Germplasm Repository, which was planted in the field for 12 months of Danzhou City, Hainan Province, China, in October 2024. All plant materials were authenticated by Dr. Zhenwen Zhang. The information of main materials and chemical agents are shown in **Table 1**.

**Table 1:** The lists of materials for experiment.

Name	Serial number	Company
DEAE-cellulose	DE-52, C3980	Beijing Solarbio Science & Technology Co., Ltd, Beijing, China
Sephadex G-200	S9160	Beijing Solarbio Science & Technology Co., Ltd, Beijing, China
LPS	L8880	Beijing Solarbio Science & Technology Co., Ltd, Beijing, China
Anti-ZO-1 antibody	TA5145S	Abmart Shanghai Co., Ltd, Shanghai, China
Anti-Occludin antibody	TB2814S	Abmart Shanghai Co., Ltd, Shanghai, China
Anti-Claudin-1 antibody	TA5364S	Abmart Shanghai Co., Ltd, Shanghai, China
Medium-temperature α-amylase	A890337	Shanghai Macklin Biochemical Technology Co., Ltd, China
Cy	PHR1404	Sigma-Aldrich, Inc., USA
RAW264.7	91062702	Sigma-Aldrich, Inc., USA
LAL Endotoxin Assay Kit	C0276S	Beyotime Biotechnology Co., Ltd, Shanghai, China
IL-2 ELISA Kit	P1575	Beyotime Biotechnology Co., Ltd, Shanghai, China
IL-4 ELISA Kit	P1612	Beyotime Biotechnology Co., Ltd, Shanghai, China
IL-6 ELISA Kit	P1326	Beyotime Biotechnology Co., Ltd, Shanghai, China
IL-10 ELISA Kit	P1522	Beyotime Biotechnology Co., Ltd, Shanghai, China
TNF-α ELISA Kit	PT512	Beyotime Biotechnology Co., Ltd, Shanghai, China

All other reagents used in this study were of analytical grade and obtained from standard commercial sources. Specific pathogen-free (SPF) male BALB/c mice (6 weeks old, body weight: 24.5 ± 1.2 g) were supplied by Taikang Medical Testing Service Hebei Co., Ltd. (Animal Use License No.: SYXK (Ji) 2021-006). A total of 32 mice were housed in an SPF-grade barrier environment with controlled environmental conditions (temperature: 20 ± 2 °C, humidity: 55 ± 10%, 12 h light/dark cycle) with ad libitum access to standard rodent chow and water.

All animal experiments were approved by the Institutional Animal Care and Use Committee (IACUC) of the Chinese Academy of Tropical Agricultural Sciences (approval No. CATAS-202403128, dated 12 March 2024) and performed in accordance with the ARRIVE guidelines (24), the China National Guidelines for Experimental Animal Welfare (GB/T 35892-2018), and the UK Animals (Scientific Procedures) Act 1986. Every effort was made to minimize animal suffering, including adherence to aseptic techniques during procedures and the use of humane endpoints.

### 2.2 Extraction of CPCR

The extraction of CPCR was performed using an optimized protocol based on previously reported methods (25). Briefly, fresh cassava pulp was homogenized and mixed with deionized water (Millipore, Bedford, MA, USA) at a solid-to-liquid ratio of 1:2.5 (w/v), followed by the addition of medium-temperature α-amylase (5 KU/g FW, optimal pH 6.5, temperature 70 °C). The mixture was subjected to ultrasonic-assisted extraction (300 W, 70 °C) for 240 min to facilitate polysaccharide release, with enzymatic hydrolysis conducted concurrently. After centrifugation at 4000×g for 20 min, the supernatant was collected, and proteins were removed by precipitation with 4% trichloroacetic acid (TCA). The protein-free supernatant was then precipitated with 80% ethanol overnight at 4 °C, and the resulting precipitate was collected by centrifugation (4,000 × g for 20 min) and subsequently dialyzed (3.5 kDa molecular weight cutoff) against deionized water for 72 h with regular water changes to eliminate low-molecular-weight impurities. The retentate was concentrated under reduced pressure (50 °C, 0.1 MPa) and lyophilized to obtain CPCR.

### 2.3 Purification of CPCR

CPCR was further fractionated through sequential chromatographic separation. Initially, anion-exchange chromatography was performed using a DEAE-cellulose DE-52 column eluted with a linear gradient of 0-0.5 M NaCl in deionized water, followed by molecular size fractionation on a Sephadex G-200 gel filtration column. Elution profiles were monitored using the phenol-sulfuric acid method (26), with continuous detection at 490 nm. Fractions containing polysaccharides were pooled based on their elution profiles, concentrated under reduced pressure, and lyophilized to obtain purified polysaccharide fractions designated as CP1, CP2, CP3, CP4, and CP5. The endotoxin content of CPCR and purified fractions was determined using the Limulus Amebocyte Lysate (LAL) assay, and no detectable endotoxin was found.

### 2.4 Structural characterization of CPs

The molecular weight of CPs was determined by high-performance gel permeation chromatography (HPGPC) according to the method established by Wang et al. (Wyatt technology, CA, USA) (27). Monosaccharide composition analysis was performed using gas chromatography-mass spectrometry (GC-MS) (ICS 5000+, Thermo, USA). Purity assessment was conducted using UV-Vis spectroscopy (TU-1810APC, Persee, Beijing, China) to detect potential protein and nucleic acid contamination, while Fourier transform infrared spectroscopy (FT-IR) analyses (Nicolet iZ-10, Thermo, USA) were performed to characterize functional groups and structural features according to the procedures described by Mahepali et al. (28).

### 2.5 *In vivo* experiment

#### 2.5.1 Animals and housing conditions

A total of 32 mice were housed in an SPF-grade barrier environment with controlled environmental conditions (temperature: 20 ± 2 °C, humidity: 55 ± 10%, 12 h light/dark cycle) with ad libitum access to standard rodent chow and water. Mice were randomly allocated to four groups using a random number generator. Investigators were blinded during sample collection and analysis. Sample size (n=8 per group) was determined based on previous similar polysaccharide studies and power analysis. Only male mice were used, which may limit generalization to female animals.

#### 2.5.2 Treatment protocol

Following a 7-day acclimatization period, mice were randomly divided into four experimental groups (n=8 per group). 1. Normal control (NC). 2. Model control (MC). 3. Positive control (PC). 4. CPCR group. The detailed treatment protocols for each group, including the administration of reagents, treatment time, and routes, are presented in **Table 2**.

**Table 2:** Experimental animal groups and handling.

Groups	0–7 day	8–10 day	11–20 day	21 day
NC	acclimatization period	received 200 μLsterile saline injections	received 200 μL distilled water daily by oral gavage	blood collection and tissue harvesting
MC		received daily intraperitoneal injections of Cy (80 mg/kg body weight) dissolved in 200 μL sterile saline	received 200 μL distilled water daily by oral gavage	
PC		received daily intraperitoneal injections of Cy (80 mg/kg body weight) dissolved in 200 μL sterile saline	received 200 μL levamisole (40 mg/kg body weight) daily by oral gavage	
CPCR		received daily intraperitoneal injections of Cy (80 mg/kg body weight) dissolved in 200 μL sterile saline	received 200 μL CPCR (350 mg/kg body weight) daily by oral gavage	

The 8~21-day protocol comprised three phases, as following:

1. Days 8–10 induction of immunosuppression. All groups except NC received daily intraperitoneal injections of Cy (80 mg/kg body weight, dissolved in 200 μL of sterile saline),while the NC received equal-volume sterile saline injections.

2. Days 11-20, treatment phase. The NC and MC received 200 μL distilled water daily by oral gavage, while the PC and CPCR groups were administered levamisole or CPCR solution (200 μL total volume), respectively. Body weight and food intake were monitored and recorded every 3 days throughout the experimental period.

3. Day 21, endpoint. Blood collection and tissue harvesting were performed, peripheral blood was collected via the retro-orbital plexus using capillary glass melting-point tubes (0.5 mm × 100 mm, Cat. No. 24101705; West China Medical University) under anesthesia before euthanasia by cervical dislocation. Primary and secondary immune organs (spleen and thymus) were immediately weighed to calculate organ indices, and colonic contents were snap-frozen in liquid nitrogen and stored at −80 °C for subsequent microbiome analysis. Organ indices were calculated as organ weight (mg) per 100 g body weight as described by Xie et al. (29).

#### 2.5.3 Serum immunoglobulin and cytokine quantification

Blood samples were allowed to clot at room temperature for 30 min, followed by centrifugation at 3,000 × g for 15 min to obtain serum. The concentrations of IgG and cytokines (IL-2, IL-4, IL-6, IL-10, and TNF-α) in serum samples were quantitatively analyzed using commercially available ELISA kits. All assays were performed strictly according to the manufacturers’ instructions, with each sample analyzed in triplicate.

#### 2.5.4 Western blot

To evaluate changes in intestinal barrier integrity, the protein expression levels of ZO-1, Occludin, and Claudin-1 in colon tissues were determined by Western blot analysis following the method described by Li et al. (30). Briefly, colon tissue samples were homogenized in protein extraction buffer, and total protein concentrations were determined using the BCA assay. Equal amounts of protein were separated by SDS-PAGE and transferred to PVDF membranes. After blocking and incubation with primary and secondary antibodies, protein bands were visualized using enhanced chemiluminescence.

#### 2.5.5 Histopathological examination

Colon tissue samples were fixed in 10% neutral buffered formalin, processed through standard histological procedures, and embedded in paraffin. Sections (5 μm thickness) were prepared and stained with hematoxylin and eosin (H&E) for morphological evaluation. Pathological evaluation was performed according to the method of Xie et al. (29) by experienced pathologists in a blinded manner.

#### 2.5.6 16S rDNA sequencing

Genomic DNA was extracted from fecal samples using standard protocols, and 16S rDNA sequencing was performed by Guangzhou Weiyu Zhihe Biotechnology Co., Ltd., following the methodology described by Su et al. (31). Briefly, genomic DNA was extracted from fecal samples using the CretMagTM Power Soil DNA Kit(CretBiotech, China). The V3–V4 hypervariable region of the 16S rRNA gene was amplified using the forward primer 341F (5′-CCTAYGGGRBGCASCAG-3′) and reverse primer 806R (5′-GGACTACHVGGGTWTCTAAT-3′). Sequencing was performed on an Illumina MiSeq platform (Illumina, San Diego, CA, USA). Raw reads were filtered with fastp (version 0.20.0) to remove low-quality reads (Q30 < 90%) and short sequences (<50 bp). High-quality reads were merged using FLASH (version 1.2.7) and clustered into amplicon sequence variants (ASVs) using the DADA2 pipeline. Operational taxonomic units (OTUs) with 97% similarity cutoff[3, 4] were clustered using UPARSE version 7.1[3], and chimeric sequences were identified and removed. The taxonomy of each OTU representative sequence was analyzed by RDP Classifier version 2.2[5] against the 16S rRNA database (e.g. Silva v138) using confidence threshold of 0.7. Beta-diversity was assessed via principal coordinate analysis (PCoA) based on the Bray–Curtis distance matrix, and group differences were tested by PERMANOVA. Multiple comparisons were corrected using the Benjamini–Hochberg method. Raw sequencing data have been deposited in the NCBI Sequence Read Archive (SRA) under accession number PRJNA1472425.

#### 2.5.7 Short-chain fatty acid

Fecal SCFA quantification was conducted according to the gas chromatography-mass spectrometry method described by Li et al. (32). Briefly, approximately 70 mg of fecal sample was homogenized with 1 mL ultrapure water, followed by vortex mixing, ultrasonication for 30 min, and centrifugation at 12,000 × g for 10 min at 4 °C. The supernatant was filtered through a 0.22 μm membrane filter and analyzed by GC-MS (Thermo Trace 1310) equipped with an HP-INNOWAX capillary column (30 m × 0.25 mm × 0.25 μm, Agilent) and a Thermo ISQ LT mass detector. Operating conditions are as follows: The initial temperature of the column was 75 °C, and the final column temperature was 220 °C. The injector temperature was 250 °C, the injection volume was 1 µL, the carrier gas was high pure nitrogen. Standard curves were prepared using authentic SCFA standards, and concentrations were expressed as μg/g fecal wet weight.

### 2.6 *In vitro* immunomodulatory assessment

#### 2.6.1 Cell culture and treatment

RAW264.7 murine macrophage cells were cultured in Dulbecco’s Modified Eagle Medium (DMEM) supplemented with 10% heat-inactivated fetal bovine serum (FBS) and 1% Penicillin/Streptomycin under standard conditions (37 °C, 5% CO_2_ atmosphere) (CI-191CX, Jingqi, Jiangsu, China). For experimental assays, cells were seeded in 96-well plates at 1×10^5^ cells/well densities and allowed to adhere for 24 h before treatment. Subsequently, cells were treated with LPS (1.0 μg/mL) as a positive control and different concentrations of polysaccharide samples (62.5, 250.0, 1000.0 μg/mL), with culture medium serving as the negative control.

#### 2.6.2 Cell viability and proliferation

Cell viability was assessed using the Cell Counting Kit-8 (CCK-8) assay according to established protocols (32). Briefly, after 24 h treatment with CPs, CCK-8 solution was added to each well and incubated for 2 h at 37 °C. Absorbance was measured at 450 nm using a microplate reader (Multiskan FC, Thermo, USA), and cell viability was expressed as a percentage relative to the control group.

#### 2.6.3 Phagocytic activity

Macrophage phagocytic activity was evaluated using the neutral red uptake assay (33). After 24 h treatment with CPs, cells were incubated with neutral red solution for 2 h, after which the internalized dye was extracted using acidified ethanol. The optical density was measured at 540 nm, and phagocytic activity was expressed as a percentage of the control.

#### 2.6.4 Nitric oxide production

Nitric oxide (NO) production in culture supernatants was determined using the Griess method. Cell culture supernatants were collected after 24 h treatment with CPs and mixed with Griess reagent according to the manufacturer’s instructions. Absorbance was measured at 540 nm, and NO concentration was calculated using a sodium nitrite standard curve.

#### 2.6.5 Cytokine secretion

The secretion levels of pro-inflammatory and anti-inflammatory cytokines, including tumor necrosis factor-α (TNF-α), interleukin-6 (IL-6), and interleukin-10 (IL-10), were quantified in culture supernatants using enzyme-linked immunosorbent assay (ELISA) kits. All experimental procedures were performed strictly according to the manufacturers’ protocols, with samples analyzed in triplicate.

#### 2.6.6 Splenocyte proliferation

The splenocyte proliferation assay was performed to evaluate the effects of polysaccharides on lymphocyte activation, with modifications based on the method described by Li et al. (30). Under sterile conditions, mouse spleens were aseptically harvested and processed to prepare single-cell suspensions through mechanical disruption and filtration. Cell suspensions were adjusted to a density of 2×10^5^ cells/well using complete medium. To specifically induce T lymphocyte and B lymphocyte proliferation, experimental groups were stimulated with concanavalin A (ConA, 1 μg/mL) and LPS (0.1 μg/mL), respectively, for 12 hours. Following stimulation, supernatants were removed, and each well was supplemented with medium containing 10% CCK-8 solution. After incubation at 37 °C in a 5% CO_2_ atmosphere for 4 hours, optical density at 450 nm was measured using a microplate reader to evaluate cell proliferation.

### 2.7 Statistical analysis

All data were processed and analyzed using SPSS 26.0 and Origin 2021. Continuous variables are expressed as mean ± standard deviation (M ± SD). Normality of distribution and homogeneity of variance were tested using the Shapiro–Wilk test and Levene’s test, respectively. Normally distributed data with equal variance were analyzed by one-way analysis of variance (ANOVA), followed by Tukey’s *post hoc* test for multiple comparisons. Data that did not meet normality or homoscedasticity assumptions were analyzed using the non-parametric Kruskal–Wallis test, followed by Dunn’s *post hoc* test with Bonferroni correction. For multiple-group comparisons, p-values were adjusted using the Bonferroni method. The 16S rRNA sequencing data were analyzed using compositional data analysis approaches in QIIME2 and R software. Alpha diversity indices (Chao1, Shannon, Simpson) were compared using the Kruskal–Wallis test followed by Dunn’s test. Beta diversity was assessed via permutational multivariate analysis of variance (PERMANOVA). Differential microbial taxa at the phylum and genus levels were identified using analysis of composition of microbiomes (ANCOM), with adjusted p-values reported. Statistical significance was defined as *p < 0.05, **p < 0.01, ***p < 0.001 versus the normal control (NC) group, and #*p* < 0.05, ##*p* < 0.01, ###*p* < 0.001 versus the model control (MC) group.

## 3 Results

### 3.1 Changes of composition in gut microbiota

**Figure 1:**
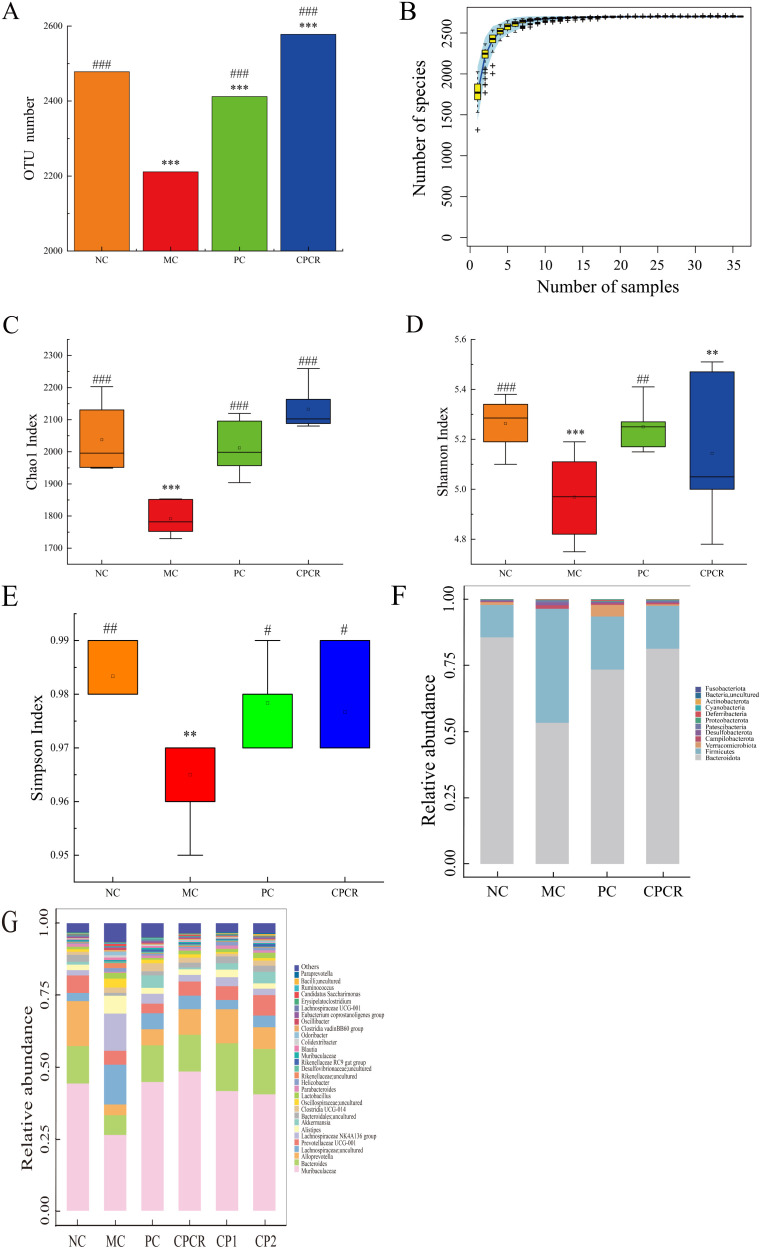
Effects of gut microbiota diversity and community composition in mice feces. **(A)** OTU number, **(B)** Observed species curve, **(C)** Chao1 index, **(D)** Shannon index, **(E)** Simpson index, **(F)** Relative abundance of microbiota at the phylum level, **(G)** Relative abundance of microbiota at the genus level. Data are expressed as mean ± SD (n = 6 mice per group). *p < 0.05, **p < 0.01 and ***p < 0.001, vs NC; ^#^*p* < 0.05, ^##^*p* < 0.01 and ^###^*p* < 0.001, vs MC.

As the body’s largest immune organ, the gut hosts a complex microbiota essential for host metabolic homeostasis and immune regulation. These microbes facilitate the metabolism of key nutrients, such as carbohydrates and proteins, and their dysbiosis is closely linked to immune dysfunction (30). In this study, we identified 1,766 operational taxonomic units (OTUs) across all experimental groups (**Figure 1A**). The CPCR group displayed a significantly elevated OTU count compared to the MC group (p < 0.001). The numbers of unique OTUs per group were 445, 712, 646, and 812 for the NC, MC, PC, and CPCR groups, respectively, with rarefaction analysis confirming adequate sequencing depth (**Figure 1B**). Alpha diversity metrics further demonstrated the efficacy of CPCR intervention. While the MC group showed a significant decline in species richness (Chao1 index), the CPCR group restored richness to the highest observed levels (**Figure 1C**). Consistently, both Shannon (**Figure 1D**) and Simpson (**Figure 1E**) indices indicated that CPCR treatment significantly enhanced microbial diversity. Notably, CPCR administration shifted the microbial community structure toward that of the NC group, indicating a potential restorative effect against Cy-induced dysbiosis.

At the phylum level, the microbiota was predominantly composed of Bacteroidota and Firmicutes (**Figure 1F**). Cy induction significantly decreased Bacteroidota abundance while increasing Firmicutes, consistent with prior findings on polysaccharide interventions (34). CPCR treatment effectively reversed these alterations by downregulating Firmicutes and upregulating Bacteroidota and *Verrucomicrobiota*. The microbial composition in CPCR-treated exhibited a normalization trend approaching that of the NC. At the genus level, The MC showed significant reductions in *Muribaculaceae*, *Bacteroides*, *Alloprevotella*, and *Prevotellaceae UCG-001*, alongside a marked increase in *Lachnospiraceae*. CPCR intervention restored the abundances of these genera to varying degrees (**Figure 1G**), with changes in *Lachnospiraceae* (Firmicutes phylum) consistent with phylum-level observations.

### 3.2 Effects of immune response

**Figure 2:**
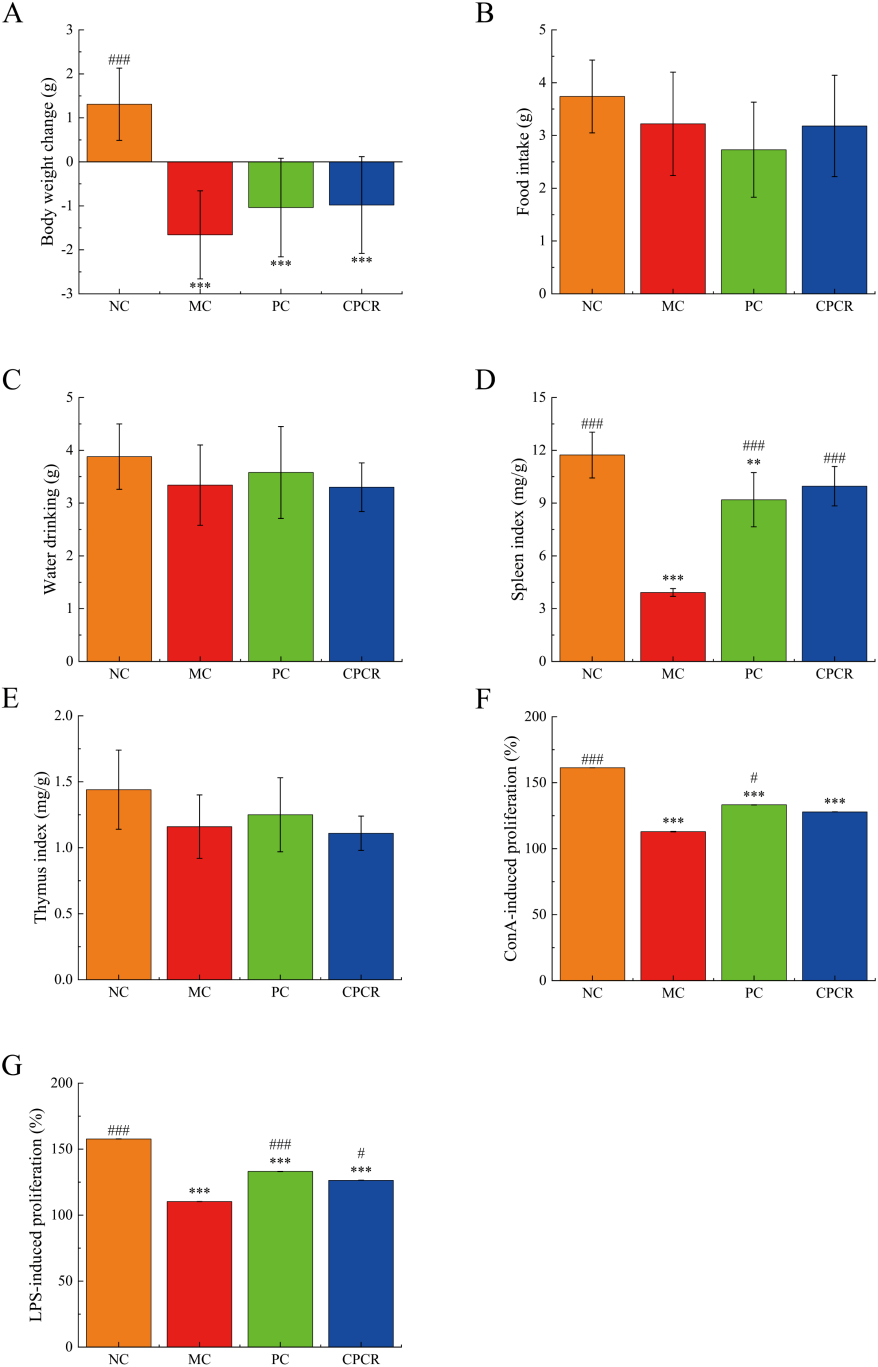
Organ indices in Cy-treated mice and proliferation rates of T/B cells in mice administered. **(A)** Body weight; **(B)** Food intake; **(C)** Water drinking; **(D)** Spleen index; **(E)** Thymus index; **(F)** ConA-induced T lymphocyte; **(G)** LPS-induced B lymphocyte. Note: NC: control group, MC: model group (Cy-treated), PC: Cy+Positive control group (levamisole 40 mg/kg/d), CPCR: Cy+CPCR group (CPCR 350 mg/kg/d). Data are expressed as mean ± SD (n = 8 mice per group). *p < 0.05, **p < 0.01 and ***p < 0.001, vs NC; ^#^*p* < 0.05, ^##^*p* < 0.01 and ^###^*p* < 0.001, vs MC.

The thymus and spleen, as central and peripheral immune hubs, are essential for immune cell maturation; thus, their organ indices serve as direct indicators of host immune status (29, 32). We observed that Cy induction caused a significant reduction in body weight (**Figure 2A**), which was effectively restored by CPCR intervention to near-baseline levels. Since no significant intergroup differences were found in food or water consumption (**Figures 2B, C**), the weight restoration by CPCR likely stems from specific immunomodulatory actions rather than improved nutritional intake. In terms of immune organ indices, Cy treatment induced marked splenic atrophy (**Figure 2D**). CPCR administration resulted in a highly significant recovery of the spleen index (*p* < 0.001 vs. MC), whereas the thymus index showed only a non-significant trend toward recovery (**Figure 2E**). This differential response suggests that CPCR predominantly targets splenic immune function. These observations align with established correlations between body weight, immune organ mass, and lymphocyte proliferative capacity.

As a hallmark of immune competence, lymphocyte proliferation is fundamental to both cellular and humoral immunity (30). ConA- and LPS-stimulated assays revealed significantly suppressed T-cell and B-cell viability in the MC group compared to the NC group (**Figures 2F, G**). Notably, CPCR treatment significantly reversed this suppression, promoting both T and B lymphocyte proliferation (*p* < 0.05). In summary, CPCR mitigates Cy-induced immunosuppression by reversing weight loss and splenic atrophy through the enhancement of splenic lymphocyte proliferation. Given the intricate crosstalk between lymphocyte proliferation and inflammatory mediator release, these results provide a foundation for exploring CPCR’s anti-inflammatory mechanisms.

### 3.3 Changes of cytokine profiles

**Figure 3:**
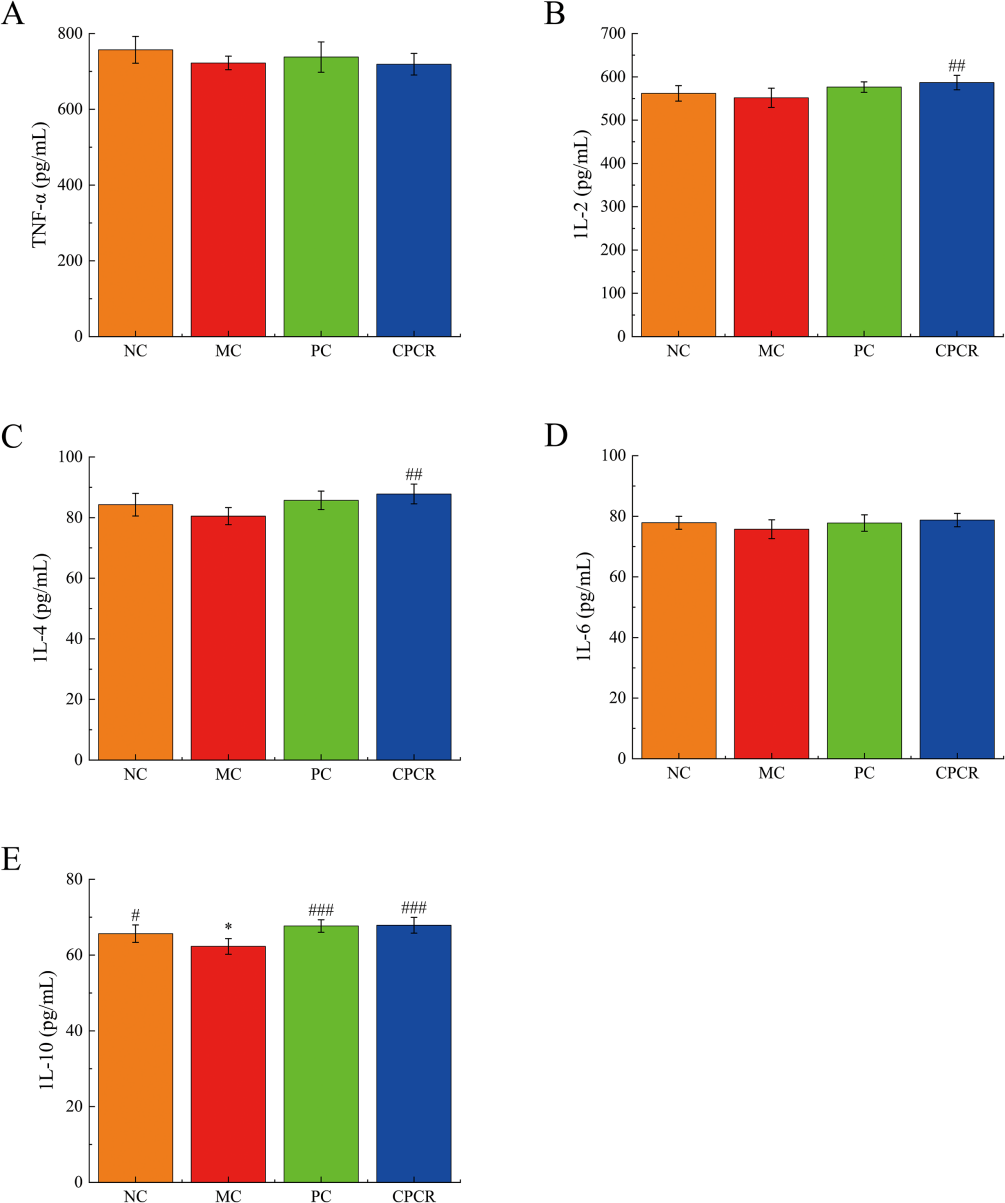
CPCR enhanced cytokine secretion in Cy-induced mice. **(A)** TNF-α; **(B)** IL-2; **(C)** IL-4; **(D)** IL-6; **(E)** IL-10. Data are expressed as mean ± SD (n = 8 mice per group). *p < 0.05, **p < 0.01 and ***p < 0.001, vs NC; ^##^*p* < 0.05, ^#^*p* < 0.01 and ^###^*p* < 0.001, vs MC.

In terms of cytokine profiles, levels of the pro-inflammatory markers TNF-α and IL-6 remained unchanged across groups (**Figures 3A, D**). In contrast, CPCR intervention markedly restored the secretion of anti-inflammatory cytokines (IL-2, IL-4, and IL-10) relative to the MC group (**Figures 3B, C, E**). This selective upregulation underscores the immunorestorative capacity of CPCR. Collectively, our findings indicate that CPCR alleviates Cy-induced immunosuppression by enhancing the production of critical immunoregulatory cytokines, including IL-2, IL-4, and IL-10.

### 3.4 CPCR restores intestinal barrier integrity and ameliorates colonic tissue damage

**Figure 4:**
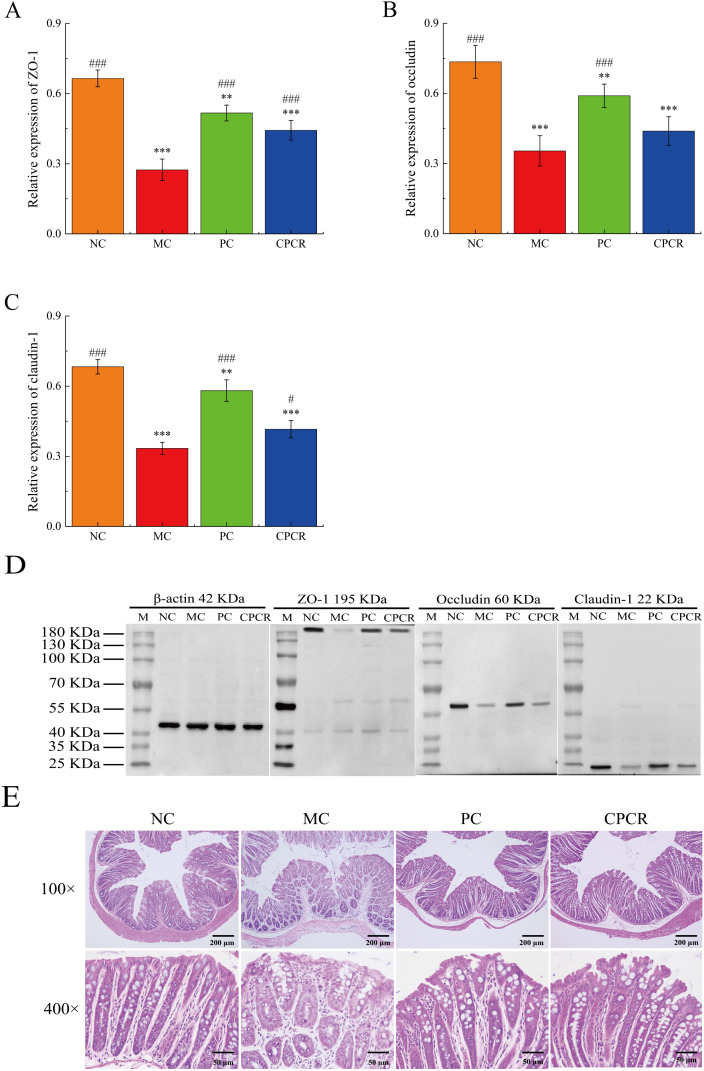
Effects of CPs on tight junction protein expression of colonic tissues and colonic histopathology. **(A)** ZO-1, **(B)** Occludin, **(C)** Claudin-1, **(D)** WB, and **(E)** H&E staining of colonic tissues (scale bar = 200 μm and scale bar = 50 μm). Western blot data are representative of 3 biological replicates, with Actin as the loading control and molecular weight markers indicated. Histological scoring was performed in a blinded manner (n = 3). Data are expressed as mean ± SD. *p < 0.05, **p < 0.01 and ***p < 0.001, vs NC; ^#^*p* < 0.05, ^##^*p* < 0.01 and ^###^*p* < 0.001, vs MC.

The intestinal barrier, critically maintained by tight junction proteins such as ZO-1, Claudin-1, and Occludin, serves as a primary defense against pathogen translocation and subsequent systemic inflammation (16). As demonstrated in **Figures 4A–D**, Cy induction significantly downregulated the expression of ZO-1, Occludin, and Claudin-1(*p* < 0.001), consistent with compromised barrier integrity and heightened intestinal permeability. Notably, CPCR intervention markedly restored the expression levels of these TJ proteins, indicating a potent restorative effect on intestinal mucosal integrity.

Concurrently, histopathological examination of cecal tissues provided structural corroboration of the protective efficacy of CPCR (35). While the NC group exhibited an intact epithelial lining with well-organized villi and dense tissue architecture, the MC group displayed severe pathological alterations, characterized by mucosal disruption and disorganized tissue structure (**Figure 4E**). In contrast, mice treated with CPCR presented histological features comparable to those of the NC group, featuring preserved epithelial continuity, regularly arranged villi, and compact tissue organization. These observations suggest that CPCR effectively mitigates Cy-induced colonic injury.

Collectively, these findings demonstrate that CPCR not only upregulates the expression of tight junction proteins to reinstate intestinal barrier function but also facilitates the structural repair of colonic tissues. This dual protective mechanism offers critical insights into the therapeutic potential of CPs in alleviating intestinal damage within immunosuppressed models. Studies have shown that changes in cytokines are indeed influenced by the metabolites of polysaccharides, with the mechanism heavily dependent on the increased production of intestinal SCFAs.

### 3.5 Changes of short-chain fatty acids

**Figure 5:**
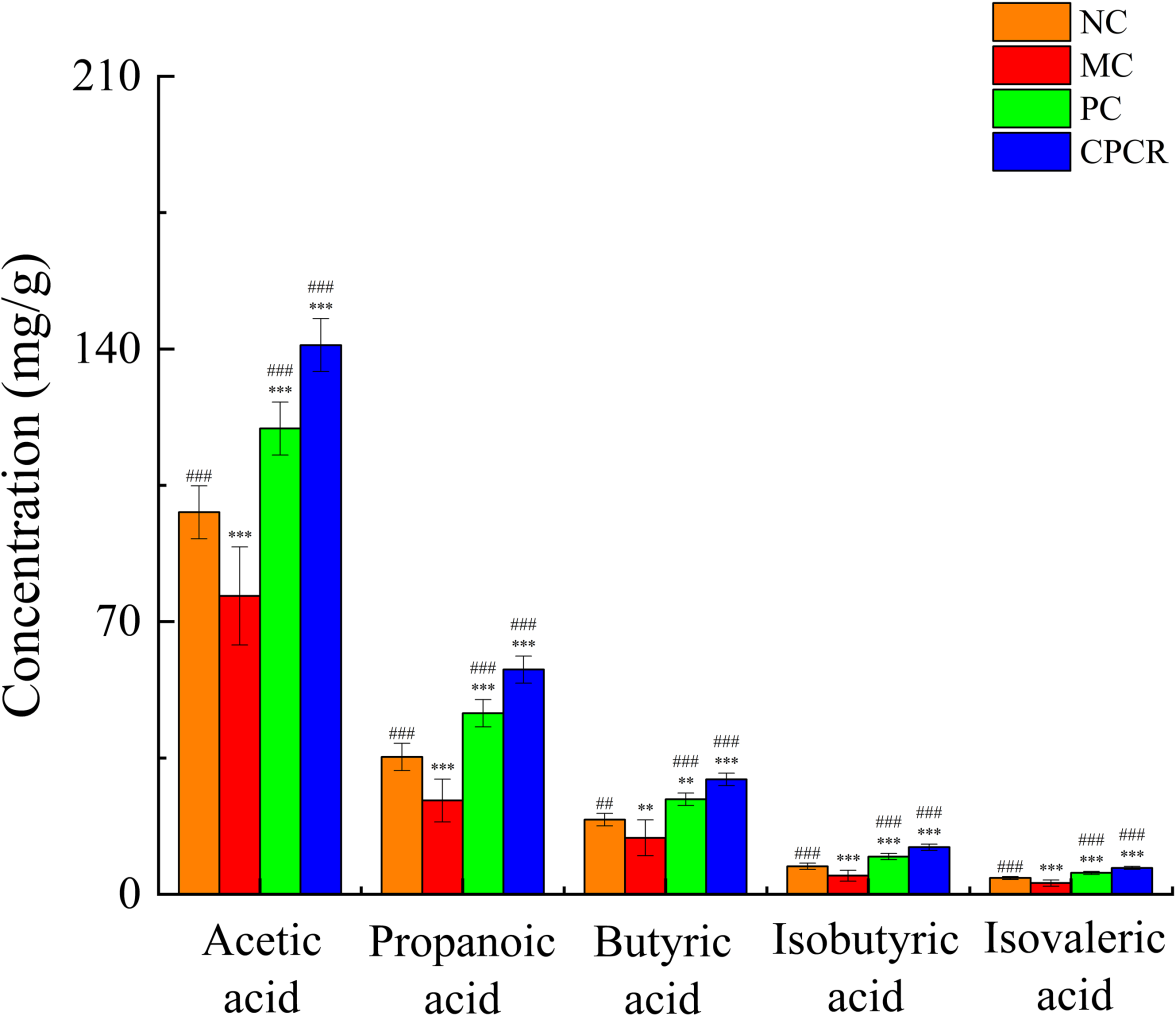
The effects of CPCR on secretion levels of SCFAs in mice feces. Data are expressed as mean ± SD (n = 6 mice per group). *p < 0.05, **p < 0.01 and ***p < 0.001, vs NC; ^#^*p* < 0.05, ^##^*p* < 0.01 and ^###^*p* < 0.001, vs MC.

SCFAs, essential microbial metabolites derived from the fermentation of complex carbohydrates, play pivotal roles in maintaining intestinal barrier integrity and immune homeostasis (16). As depicted in **Figure 5**, Cy induction led to a significant reduction (*p* < 0.01) in the levels of all detected SCFAs compared to the NC group, indicating a severe impairment of microbial metabolic activity consequent to immune injury. Notably, CPCR intervention not only completely reversed the Cy-induced depletion of SCFAs but also elevated their concentrations to levels significantly exceeding those observed in the NC group (p < 0.001). This supra-physiological enhancement may be attributed to the capacity of SCFAs to lower intestinal pH, thereby creating a favorable microenvironment for probiotic proliferation. Specifically, after CPCR treatment, the concentration of acetic acid was significantly higher than that in the control and Cy-induced injury groups, reaching 141.0 mg/g feces. This process likely establishes a positive feedback loop that amplifies SCFA production within immunocompromised hosts (32).These metabolic findings are consistent with the previously observed increase in the abundance of Bacteroidota (**Figure 5**), a phylum known to encompass key SCFAs-producing taxa. Collectively, our results demonstrate that CPCR supplementation effectively restores SCFAs levels and revitalizes microbial metabolic function in the intestine. Accordingly, we further investigated the *in vitro* immunomodulatory effects of CPCR.

### 3.6 Characterization of CPCR

**Figure 6:**
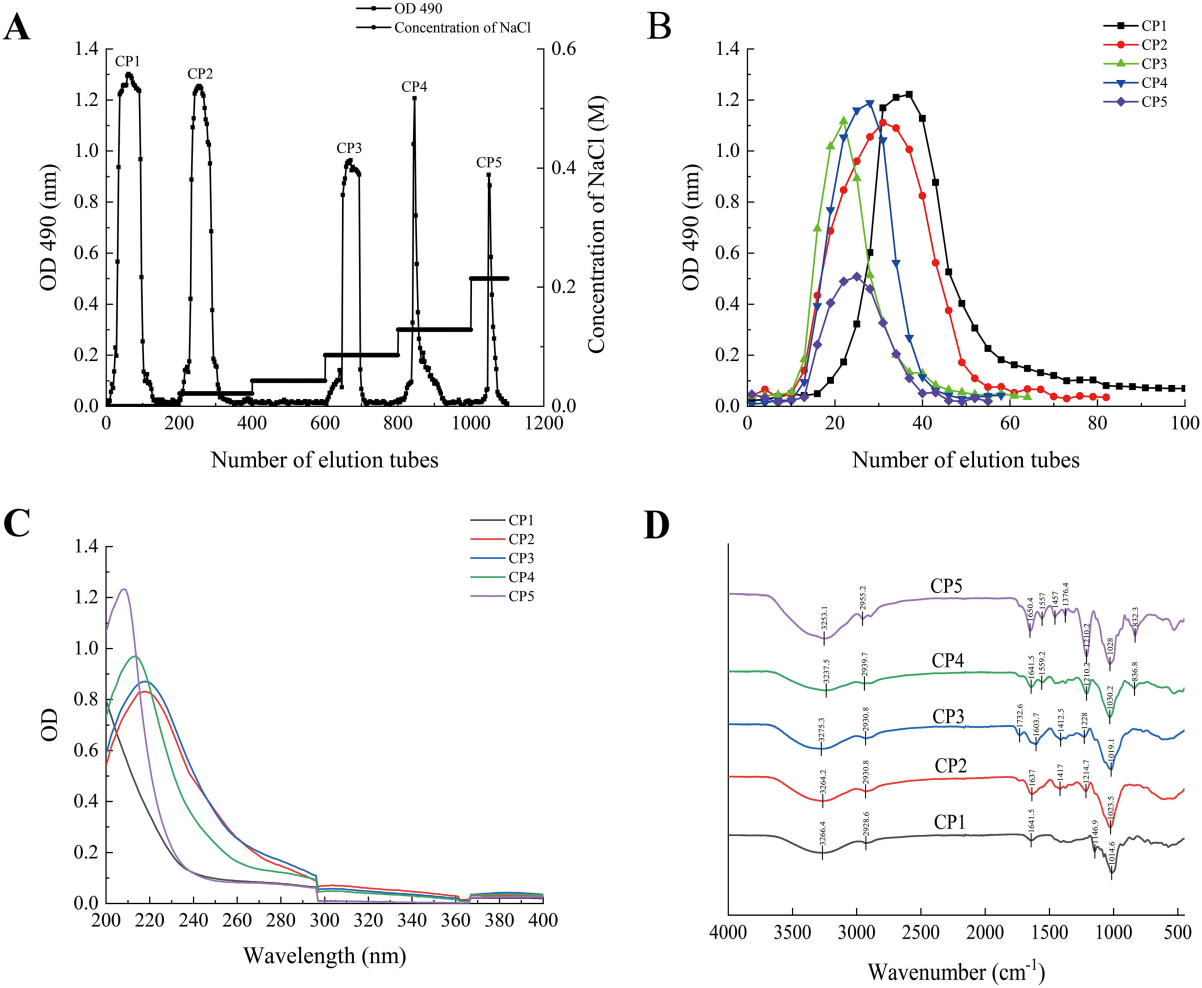
Purification and structural characterization of Polysaccharides from cassava root tubers. **(A)** Elution profile of the CPs on DEAE-Sephacel column; **(B)** Elution profile of CP1-CP5 on Sephadex G-200 column; **(C)** UV-vis spectrum; **(D)** FT-IR spectrum.

The elution profiles of polysaccharides isolated from cassava roots are presented in **Figure 6**. Chromatographic purification of CPs from cassava roots yielded five well-resolved fractions (**Figure 6A**), designated as CP1, CP2, CP3, CP4, and CP5. Each fraction exhibited a single, symmetrical elution peak, indicative of high homogeneity. The yield distribution (**Figure 6B**) revealed a descending order of abundance: CP1 > CP2 > CP3 > CP4 > CP5. These results are consistent with established protocols for polysaccharide fractionation, thereby validating the reliability and efficacy of the isolation strategy employed in this study (15).

Structural characterization (**Table 3**) revealed distinct physicochemical properties among the five fractions (CP1-CP5). CP1 and CP5 were identified as neutral polysaccharides, composed predominantly of arabinose and glucose. In contrast, CP2 through CP4 were characterized as acidic polysaccharides, containing significant amounts of uronic acids and exhibiting heterogeneous monosaccharide compositions. The molecular weights (Mw) of these acidic fractions ranged from 36.9 to 62.4 kDa. Notably, CP1 exhibited the highest glucose content (97.0%) and the lowest molecular weight (3.0 kDa), whereas CP2 possessed the highest mannose content (5.6%) and the largest molecular weight (62.4 kDa), which was approximately 20.8-fold greater than that of CP1. The purified fractions were further characterized using UV-Vis and FT-IR (**Figures 6C, D**). UV-Vis spectra showed no distinct absorption peaks at 260 nm or 280 nm for any fraction, confirming the absence of nucleic acids and proteins and indicating high purity. The FT-IR spectra displayed characteristic polysaccharide absorption bands (**Figure 6D**): a broad band at ~3400 cm^-1^ attributed to O-H stretching vibrations, a peak at ~2930 cm^-1^ corresponding to C-H stretching, and signals at 1148 cm^-1^ (C-O-C glycosidic linkage) and 1014–1030 cm^-1^(pyranose ring vibrations). Furthermore, specific absorption features in the fingerprint region suggest the presence of α-glycosidic configurations within these polysaccharide structures.

**Table 3:** Monosaccharide composition and molecular weight of CP1–CP5.

Sample	Content (mol, %)									Mw (KDa)
	Fucose	Arabinose	Rhamnose	Galactose	Glucose	Xylose	Mannose	Galacturonic acid	Glucuronic acid	
CP1	0.0^c^	2.3^e^	0.0^d^	0.8^d^	97.0^a^	0.0^d^	0.0^d^	0.0^d^	0.0^c^	3.0^e^
CP2	3.1^b^	15.2^d^	8.8^c^	20.0^c^	31.1^c^	10.7^b^	5.6^a^	3.2^c^	2.5^b^	62.4^a^
CP3	7.8^a^	18.2^c^	14.4^a^	25.6^b^	3.8^e^	13.6^a^	2.7^c^	10.1^a^	3.9^a^	42.7^b^
CP4	3.0^b^	21.6^b^	11.2^b^	28.8^a^	12.3^d^	10.0^c^	5.4^b^	3.8^b^	3.9^a^	36.9^c^
CP5	0.0^c^	35.4^a^	0.0^d^	0.0^e^	64.6^b^	0.0^d^	0.0^d^	0.0^d^	0.0^c^	14.1^d^

Different superscript letters a, b, c, d and e indicate significant (*p* < 0.05) differences between samples in each group.

### 3.7 Immunomodulatory effects of CPs *in vitro*

**Figure 7:**
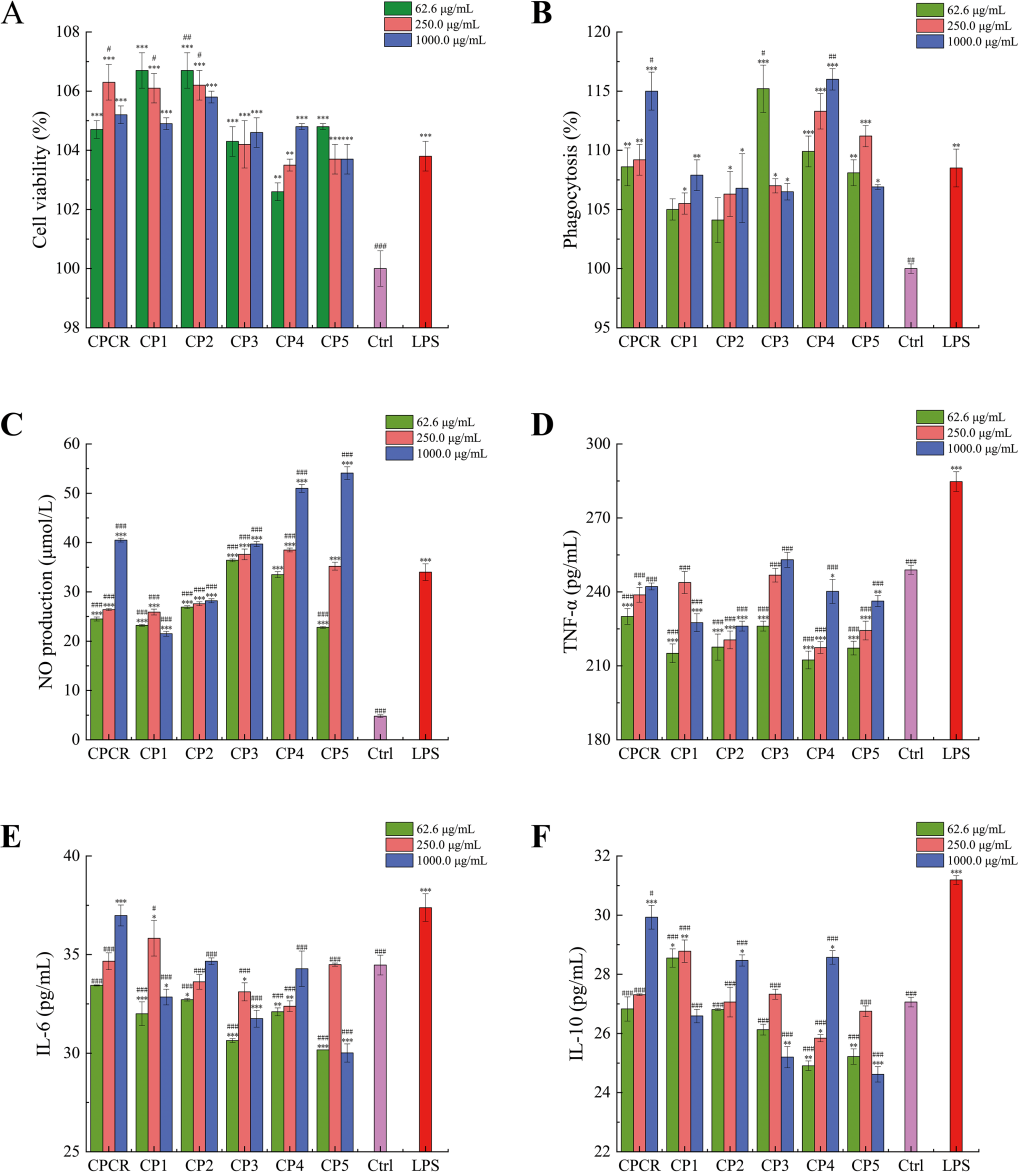
Effects of CP1-CP5 on immunomodulatory activity. **(A)** Cell viability; **(B)** Phagocytosis; **(C)** NO production; **(D)** TNF-α secretion; **(E)** IL-6 secretion; **(F)** IL-10 secretion. Data are presented as means ± SD (n = 3 independent experiments). *p < 0.05, **p < 0.01 and ***p < 0.001, vs control, ^#^*p* < 0.05, ^##^*p* < 0.01 and ^###^*p* < 0.001, vs LPS.

The immunomodulatory potential of CPs was systematically evaluated using *in vitro* assays with RAW264.7 macrophages. Both crude polysaccharides from CPCR and the purified CP fractions significantly promoted macrophage proliferation at concentrations of 62.5, 250.0, and 1000.0 μg/mL, without exhibiting cytotoxicity; cell viability remained consistently above 100% relative to the control (**Figure 7A**). Notably, the CP2 fraction exhibited the most pronounced proliferative effect at 62.5 μg/mL, increasing cell viability to 106.7%. Beyond proliferation, the phagocytic capacity of macrophages—a cornerstone of innate immune defense (32)—was remarkably enhanced by all CP fractions compared to the control (**Figure 7B**). Specifically, CP4 demonstrated the most potent pro-phagocytic activity at 1000.0 μg/mL, achieving a phagocytosis rate of 116.0%, which significantly surpassed even the lipopolysaccharide (LPS)-positive control. Similarly, the level of nitric oxide (NO) is also an important indicator of immune regulation, a critical mediator of macrophage effector functions—in a concentration dependent manner (32). Our results revealed that CPs stimulated NO production (**Figure 7C**), except for CP1, which is no concentration dependence, may be due to its simple monosaccharide composition. Regarding cytokine secretion, while none of the CP fractions significantly induced tumor necrosis factor-alpha (TNF-α) release across the tested concentrations (**Figure 7D**), they differentially modulated other key cytokines. Specifically, both CPCR and CP1 significantly increased interleukin-6 (IL-6) secretion (**Figure 7E**). Moreover, CPCR, CP1, CP2, and CP4 markedly elevated levels of the anti-inflammatory cytokine interleukin-10 (IL-10) (**Figure 7F**). Collectively, these findings indicate that all CPs fractions enhance macrophage immunocompetence, with CP2 displaying the most robust overall activity *in vitro*. Based on these superior immunomodulatory properties, subsequent structural characterization was prioritized to elucidate the compositional basis of these bioactivities.

## 4 Discussion

Natural polysaccharides have garnered substantial attention as promising immunomodulatory agents owing to their multi-dimensional regulatory capabilities and favorable biocompatibility profiles (2, 7). Cassava (*Manihot esculenta* Crantz), a globally pivotal food staple crop, is endowed with abundant non-starch polysaccharides that possess considerable untapped bioactive potential (18, 19). In this study, we found through mouse experiments that CPCR can significantly improve the gut microbiota and enhance immune regulatory functions. This regulation is closely related to the composition of polysaccharides, especially CP2, which has a molecular weight of 62.4 KDa and exhibits the best activity.

### 4.1 *In vivo* immunomodulatory activities

Cy-induced immunosuppression is characterized by compromised immune cell functionality, disrupted intestinal barrier integrity, and gut microbiota dysbiosis (24, 34). Our results demonstrated that CPCR administration effectively reversed Cy-induced weight loss and splenic atrophy, promoted the proliferation of T and B lymphocytes. These findings indicate that CPCR potentiates both cellular and humoral immune responses, which is consistent with the immunomodulatory effects of plant-derived polysaccharides such as Dendrobium officinale leaf polysaccharides and Astragalus polysaccharides (29, 30). Notably, CPCR specifically upregulated the secretion of anti-inflammatory cytokines (interleukin IL-2, IL-4, IL-10) without exerting a significant impact on the production of pro-inflammatory tumor necrosis factor-α (TNF-α). This balanced regulatory pattern, which circumvents excessive inflammatory responses, represents a critical advantage for potential translational applications (15).

The gut-immune axis plays a pivotal role in systemic immune homeostasis, and the integrity of the intestinal barrier is indispensable for preventing microbial translocation and subsequent systemic inflammation (16, 24). Our study revealed that CPCR significantly upregulated the expression of tight junction proteins (ZO-1, Occludin, Claudin-1) and mitigated Cy-induced colonic tissue lesions, thereby effectively restoring intestinal barrier function. Concurrently, 16S rDNA sequencing analysis demonstrated that CPCR modulated gut microbiota composition by increasing the relative abundance of Bacteroidota and decreasing that of Firmicutes, consequently reducing the Firmicutes/Bacteroidota (F/B) ratio—a shift closely associated with improved metabolic and immune homeostasis (15, 34). Furthermore, CPCR significantly elevated the levels of fecal SCFAs, including acetate, propionate, and butyrate. As key metabolites of gut microbiota, SCFAs serve as primary energy sources for colonocytes, reinforce the intestinal barrier, and inhibit pro-inflammatory signaling pathways via the nuclear factor-κB (NF-κB) cascade (36, 37). However, we acknowledge that the current inflammatory and immune profiling remains limited. Additional comprehensive analyses, including more cytokines, chemokines, and immune cell phenotyping, are required to fully validate and elaborate the precise gut–immune axis mechanism.

### 4.2 Structural characteristics and *in vitro* immunostimulatory activity

Purification of CPCR yielded five distinct fractions (CP1–CP5) with divergent structural features. Among these, CP2 exhibited the highest molecular weight (62.4 kDa) and a heterogeneous monosaccharide composition enriched in mannose and uronic acids. Previous studies have established that polysaccharides with higher molecular weights and complex monosaccharide compositions typically exhibit enhanced bioactivity, attributed to their superior capacity to interact with immune cell receptors (27). FT-IR confirmed that CP2 possesses typical α-configured polysaccharide structural motifs (e.g., pyranose rings, C-O-C linkages), This preliminary structural information suggests that CP2 may interact with Toll-like receptor 4 (TLR4) on macrophages, potentially triggering downstream immune activation (14, 15).

*In vitro* experiments utilizing RAW264.7 macrophages demonstrated that all CPs fractions enhanced macrophage proliferation, phagocytic capacity, and NO production, with CP2 exhibiting the most potent effects. Nitric oxide is a key mediator of macrophage-mediated immune responses, and its production is closely associated with innate immune activation (32). Additionally, CPCR, CP1, CP2, and CP4 significantly elevated IL-10 secretion, while CPCR and CP1 increased IL-6 levels. These results indicate that CPs modulate macrophage function by regulating cytokine secretion, which is consistent with the immunomodulatory mechanisms of sulfated Cyclocarya paliurus polysaccharides and fucoidan (14, 35). The differential bioactivities among the fractions may be attributed to structural disparities: CP1, characterized by a low molecular weight (3.0 kDa) and high glucose content, exhibited weaker concentration-dependent NO production, whereas CP2 and CP4, with more complex structural features, displayed robust cytokine-modulating effects. This observation underscores the structure-activity relationship of CPs. Notably, CP2 showed the higher yield among all fractions and significantly enhanced IL-10 secretion *in vitro*, highlighting its advantages as a promising bioactive candidate.

### 4.3 Comparative advantages and novelty

In contrast to previous research on CPs, which primarily focused on anti-fatigue, antioxidant, and hepatoprotective effects (20–22), this study is the first to systematically explore their immunomodulatory mechanisms through an integrated approach combining *in vivo* and *in vitro* experiments. The innovative “*in vivo* efficacy first” research design confirms the biological activity of CPCR, providing a translational foundation for the development of functional foods utilizing crude polysaccharides—an approach with significant industrial application potential due to its low production cost and high availability.

Furthermore, this study provides correlative evidence that CPs are associated with modulate of the gut microbiota-SCFAs-intestinal barrier axis, expanding the current understanding of their immunomodulatory pathways. While polysaccharide-mediated gut microbiota regulation has been documented for other botanical sources (24, 30), our study specifically links CPCR administration to alterations in Bacteroidota, Firmicutes and *Prevotellaceae* abundance, which activates GPR43/41 receptors by producing SCFAs inhibits the NF-κB pathway, thereby exerting anti-inflammatory effects (38). Additionally, the identification of CP2 as a key bioactive fraction with a high molecular weight and enriched mannose/uronic acid content provides preliminary insights into the potential structure-activity relationship of CPs.

**Figure 8:**
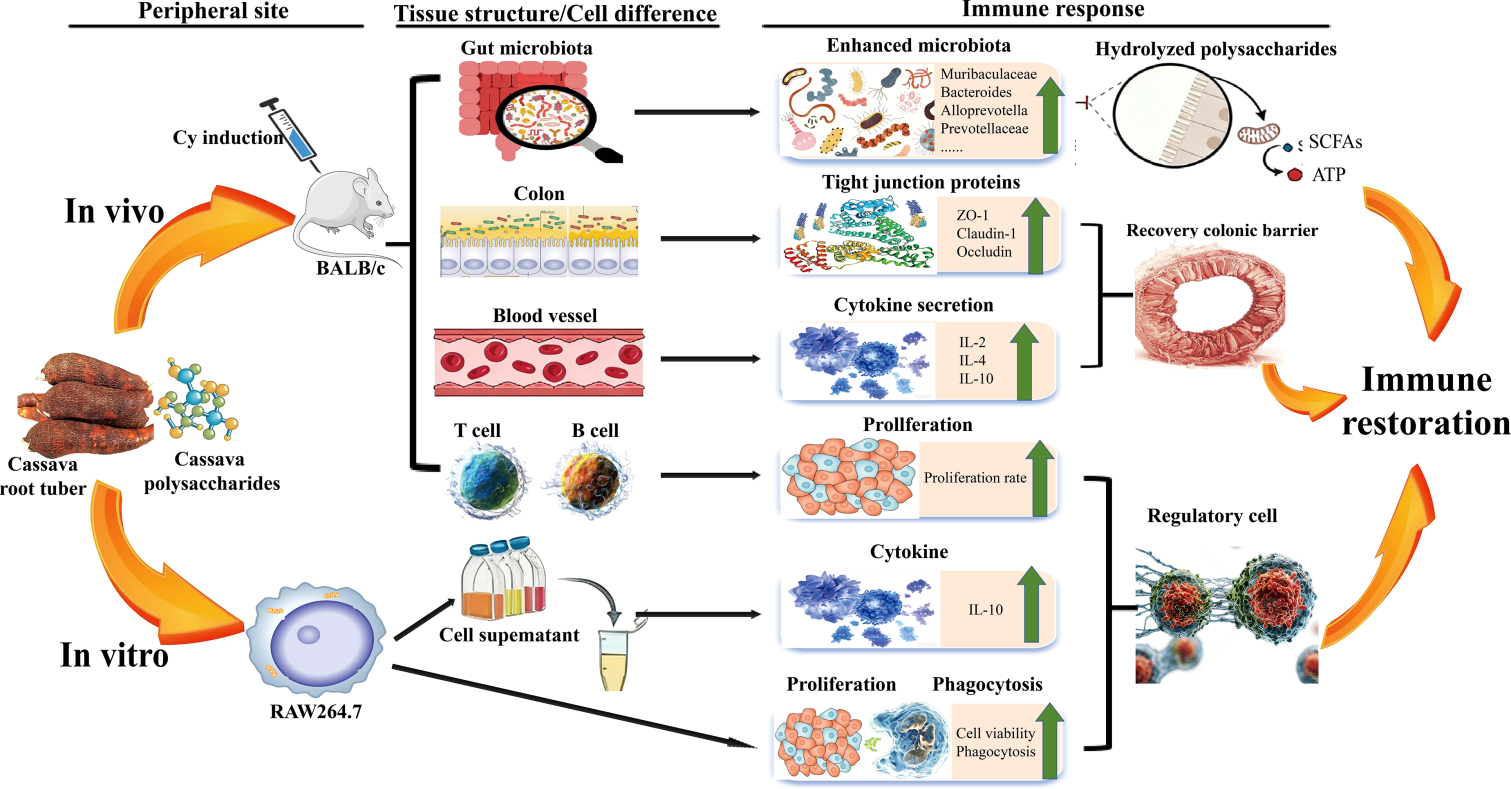
Mechanism of CPs in regulating immunity.

Based on our comprehensive findings, we propose that CP2 may contribute to immune regulation via an integrated network involving both direct and indirect pathways (**Figure 8**). Direct effects include the activation of macrophages and enhancement of lymphocyte function, while indirect effects involve the restoration of gut barrier integrity, modulation of microbial composition, and enhancement of beneficial microbial metabolites. These findings highlight the potential of CPs as natural immunomodulatory agents for functional food or pharmaceutical development, providing a scientific basis for the comprehensive utilization of cassava resources.

## 5 Conclusions

This study explored the immunomodulatory effects, mechanisms of CPs and CPCR. Results confirm CPCR exerts significant *in vivo* and *in vitro* immunomodulatory effects. *In vivo*, CPCR regulates gut microbiota by modulating beneficial commensal bacteria (*Muribaculaceae*, *Bacteroides*, *Alloprevotella*, *Prevotellaceae*), increasing intestinal SCFAs. It also upregulates intestinal tight junction proteins (ZO-1, occludin, Claudin-1) to restore intestinal barrier function and modulates inflammatory balance by increasing anti-inflammatory cytokines (IL-2, IL-4, IL-10), without altering pro-inflammatory cytokines (IL-6, TNF-α). Preliminary chemical characterization and *in vitro* verification identified five CPCR fractions (CP1-CP5). All fractions enhance RAW264.7 macrophage activity (phagocytosis, IL-10 cytokine secretion), with CP2 showing the strongest effect. In summary, CPs exert immunomodulatory effects *in vivo* and *in vitro*. CP2, with high yield and potent IL-10-inducing activity *in vitro*, represents a promising candidate for further investigation. This study provides correlative insights into the immunomodulatory effects of CPs, provides a theoretical basis for their development, and confirms their potential as functional food ingredients with immunomodulatory potential.

## Data Availability

The original contributions presented in the study are included in the article/**Supplementary Material**. Further inquiries can be directed to the corresponding author.
