# Supplementary material for: Effects of cassava polysaccharides on gut microbiome, intestinal barrier and macrophage activation
Source: Front Immunol. 2026 Jul 2;17:1874777. doi: 10.3389/fimmu.2026.1874777 (PMC13372648; doi:10.3389/fimmu.2026.1874777)
Supplement: Supplementary file 1 [file Supplementaryfile1.docx]

### **Endotoxin contents in six cassava polysaccharide samples**

**Assay kit:** LAL Endotoxin Assay Kit (Beyotime Biotechnology, C0276S, Shanghai, China), **Detection limit:** 0.01 EU/mL

#### Table S1 Standard curve data

| **Endotoxin (EU/mL)** | **OD_545_ (Replicate 1)** | **OD_545_ (Replicate 2)** | **OD_545_ (Replicate 3)** | **Mean OD_545_** |
| --- | --- | --- | --- | --- |
| 0.000 | 0.041 | 0.043 | 0.042 | 0.042 |
| 0.010 | 0.129 | 0.137 | 0.139 | 0.135 |
| 0.025 | 0.265 | 0.271 | 0.268 | 0.268 |
| 0.050 | 0.508 | 0.522 | 0.506 | 0.512 |
| 0.075 | 0.718 | 0.725 | 0.720 | 0.721 |
| 0.100 | 0.957 | 0.965 | 0.964 | 0.962 |

**Regression equation**:

X ( EU/mL) =$\frac{\mathrm{OD}545 - 0.0427}{9.168}$, R² = 0.9996

Table S2 Quality control results

| **Sample / Control** | **OD_545_** | **Endotoxin**  **(EU/mL)** | **Recovery**  **(%)** | **Pass**  **(50–200%)** |
| --- | --- | --- | --- | --- |
| Negative control (BET water) | 0.042 | < 0.01 | – | Yes |
| Positive control (0.1 EU/mL) | 0.958 | 0.096 | 0.96 | Yes |
| CPCR spike‑recovery (spiked with 0.1 EU/mL) | 0.931 | 0.093 | 0.93 | Yes |
| CPCR + polymyxin B (10 μg/mL) | 0.043 | < 0.01 | – | Yes |

Table S3 Endotoxin levels in six CP samples

| **Sample** | **Replicate 1 OD_545_** | **Replicate 2 OD_545_** | **Replicate 2 OD_545_** | **Mean OD_545_** | **Endotoxin**  **(EU/mL)** | **Result** |
| --- | --- | --- | --- | --- | --- | --- |
| CPCR | 0.043 | 0.044 | 0.039 | 0.042 | < 0.01 | Negative |
| CP1 | 0.042 | 0.045 | 0.042 | 0.043 | < 0.01 | Negative |
| CP2 | 0.035 | 0.042 | 0.037 | 0.038 | < 0.01 | Negative |
| CP3 | 0.045 | 0.041 | 0.046 | 0.044 | < 0.01 | Negative |
| CP4 | 0.041 | 0.042 | 0.04 | 0.041 | < 0.01 | Negative |
| CP5 | 0.043 | 0.045 | 0.044 | 0.044 | < 0.01 | Negative |

**Conclusion:** All six CPs samples contain endotoxin below the detection limit (<0.01 EU/mL), and polymyxin B completely neutralizes spiked endotoxin, confirming that the assay is specific and that the samples are free from biologically relevant endotoxin contamination.
